# Breast Cancer during Pregnancy—Current Paradigms, Paths to Explore

**DOI:** 10.3390/cancers11111669

**Published:** 2019-10-28

**Authors:** Ayelet Alfasi, Irit Ben-Aharon

**Affiliations:** 1 Division of Oncology, Rambam Health Care Center, Haifa 3109601, Israel; 2 Rapport Faculty of Medicine, Technion, Haifa 3200000, Israel

**Keywords:** breast cancer, pregnancy, chemotherapy, neonatal outcomes

## Abstract

Breast cancer is the most common form of malignancy in pregnant women. The prevalence of pregnancy-associated breast cancer (PABC) is up to 0.04% of pregnancies and is expected to rise in developed countries. PABC represents a unique clinical scenario which requires a delicate balance of risks and benefits for both maternal and fetal well-being. Currently, there is paucity of data regarding the short- and long-term outcomes of in-utero exposure to anti-neoplastic agents. In general, when possible, treatment for PABC should follow the same guidelines as in non-pregnant patients. Surgery, including sentinel lymph node biopsy, is possible during all trimesters of pregnancy. Radiotherapy is contraindicated during pregnancy, although it might be considered in highly selected patients based on risk–benefit assessment. Evidence supports that administration of chemotherapy may be safe during the second and third trimesters, with cessation of treatment three weeks prior to expected delivery. Currently, hormonal therapy and anti-HER2 agents are contraindicated during pregnancy and should be postponed until after delivery. Prematurity is associated with worse neonatal and long-term outcomes, and thus should be avoided. While current data on the long-term effects of anti-neoplastic treatments are reassuring, grade of evidence is lacking, hence additional large prospective studies with long-term follow-up are essential to rule out any treatment-induced adverse effects.

## 1. Introduction

Breast cancer is the most common form of cancer diagnosed during pregnancy and occurs in 1 to 4 cases per 10,000 pregnancies [1,2,3]. This incidence is expected to rise due to delaying childbearing until later in life combined with increased incidence of young-onset breast cancer.

Pregnancy-associated breast cancer (PABC) is defined as breast cancer diagnosed during pregnancy or within a year after delivery. Physiological changes during pregnancy, such as breast enlargement, changes in texture and nipple discharge, may blur those of breast cancer and thus delay the diagnosis [4]. This may result in more advanced stages at diagnosis when compared to non-pregnant women, leading to poorer outcomes [5]. Moreover, estrogen-receptors (ER) positive tumors are less common in younger women [6] and are found in even lower rates in PABC patients compared with age matched controls [7,8]. Nevertheless, HER2-positive tumors are relatively more frequent in pregnant patients [7], which may potentially contribute to poorer prognosis; however, similar overall survival (OS) and disease-free survival (DFS) were reported when comparing PABC to non-pregnant breast cancer patients when adjusted for age at diagnosis, stage, grading, histologic tumor type, receptor status, and treatment type [7,9].

A large population-based cohort retrospective study compared pregnancy outcomes between pregnant women with and without neoplasms [10]. Pregnancy-associated cancer had higher risk for preterm delivery, small for gestational age, stillbirths, and neonatal mortality. A similar study found increased risk for pregnancy-associated anemia, oligohydramnions, caesarean delivery, preterm delivery, low birth weight, infant jaundice, low Apgar score and longer neonatal hospitalizations in women with benign or malignant neoplasm [11]. However, a study including over 11 million births, among them 772 of PABC patients, found greater risk of preterm delivery and preterm premature rupture of membranes, although no association between PABC and intra-uterine growth restriction (IUGR), congenital malformations, or intrauterine fetal death was observed [12].

Former studies have addressed the effect of chemotherapy on obstetrical and neonatal outcomes concluding that immaturity may underlie neonatal sequel and thus recommend avoiding premature delivery [13]. In recent years, improvement in therapeutic modalities and the growing available data on cancer during pregnancy have led to a significant increase in pregnant patients treated with chemotherapy, resulting in a decrease in non-treated pregnant patients, a rise in birth rate of live babies, a reduction in the risk of preterm live birth and in iatrogenic preterm deliveries, leading to better outcomes [14]. In this review, we will discuss the arsenal of anti-neoplastic therapeutic tools, and the current evidence of the impact of treatment on obstetrical and neonatal outcomes.

## 2. Diagnosis

Risk factors for PABC are similar to those for age-adjusted breast cancer. Women with BRCA1 or BRCA2 mutations are at an increased risk for breast cancer at a young age, and this risk is speculated to rise even higher due to hormonal changes during pregnancy [15]. Whether the incidence of PABC is higher among mutation carriers is controversial; however, close monitoring for breast cancer during and after pregnancy is recommended.

**Figure 1:**
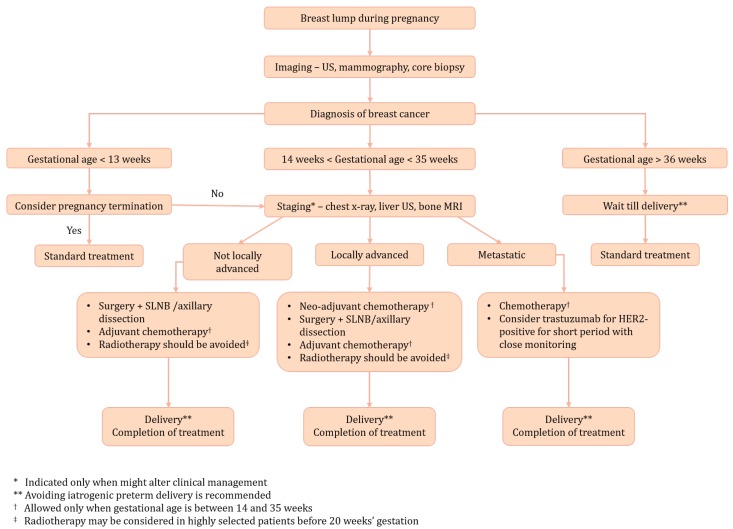
Management of pregnancy-associated breast cancer (PABC) patients.

An algorithm for the management of PABC patients is shown in Figure 1. Due to physiological changes during pregnancy which were discussed above, initial presentation in PABC patients is usually a palpable and painless lump. The initial diagnosis is made by using a breast ultrasound, as this method is considered safe and has high sensitivity and specificity [16]. In any case of suspicious mass, an ultrasound-guided core biopsy under local anaesthesia should be made immediately as histopathologic diagnosis based on core biopsy of the suspicious lesion is the gold standard for PABC. Although histological features of PABC are not different from those of young non-pregnant patients, the pathologist should be notified about the pregnancy. Invasive ductal carcinoma is the most common type of accounting for about 80–90% of cases. PABC cases have a higher prevalence of hormone–receptor (HR) negative, HER2-positive, and Ki67 positive (defined as Ki67 level > 14%) when compared to young non-pregnant breast cancer patients [17,18]. These features result in lower rates of luminal A subtype and higher prevalence of triple-negative and HER2-overexpression breast cancer subtypes in PABC patients [19].

Once a malignant lesion is confirmed by pathology, bilateral mammography is recommended to exclude bilateral and multicentric disease. Mammography is considered safe for the fetus, as a breast radiation dose is around 3 mGy, where the absorbed dose by the fetus is approximated to be less than 1 μGy [20,21], much lower than the 100 mGy threshold, which is considered safe for the fetus. Information on the safety of iodinated and gadolinium-based contrast agents during pregnancy is lacking, as gadolinium-based contrast agents are known to cross the placental barrier [22]. Therefore, a contrast-enhanced breast magnetic resonance imaging (MRI) should be avoided in PABC patients and used only in advanced stages where it may alter clinical management. If MRI is strictly indicated, approved contrast agents include gadobenate dimeglumine and gadoterate meglumine [23].

Radiographic examinations for staging purposes should be avoided during pregnancy and used only when the estimated risk of metastatic disease is high, and the results may change clinical decision. An expert team including radiologists and nuclear medicine physicians should be involved in strategy planning to evaluate and minimize fetal cumulative radiation exposure [24]. General imaging modalities used for metastatic investigations and staging in PABC patients include chest X-ray, liver ultrasound and non-contrast bone MRI, all considered safe for the fetus. The preferred method for diagnosis of bone metastases is non-contrast MRI; however, bone scintigraphy is possible only when MRI results are inconclusive or when MRI is inaccessible [23]. PET scan is not indicated in staging in non-pregnant breast cancer patients and therefore neither in PABC patients.

## 3. Treatment

**Figure 2:**
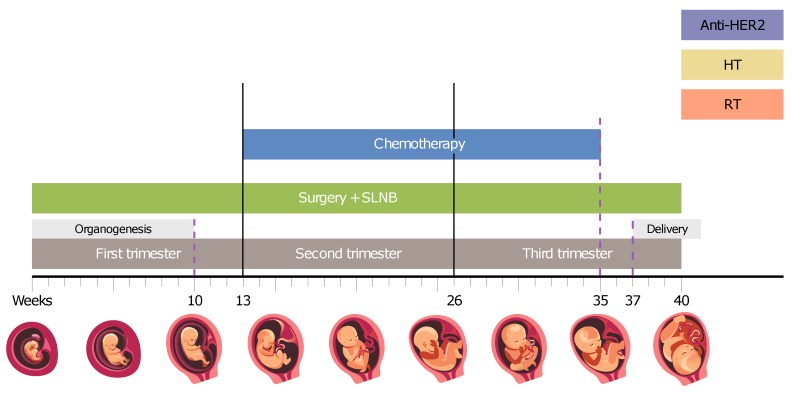
Recommended time line for various anti-neoplastic modalities. Surgery is potentially safe at all phases. Chemotherapy is recommended from week 14 (to allow a ’safe period’ after organogenesis) and should be discontinued around week 35 until after delivery to avoid myelosuppression at time of delivery. Hormonal therapy (HT), radiation therapy (RT), and targeted therapy (anti-HER2) should be postponed until after delivery. SLNB—sentinel lymph node biopsy.

Figure 2 elaborates on the various anti-neoplastic modalities and the potential application during pregnancy based on up-to-date data.

### 3.1. Surgery

The surgical approach is considered safe at any stage of pregnancy (see Figure 2) and surgical consideration should resemble those of non-pregnant patients [4,25]. Several studies found most anaesthetic agents to be safe for the fetus [26,27].

Unlike previously presumed, pregnancy alone is not an indication for mastectomy, and breast conserving surgery may be safely performed in most cases [28]. In the second and third trimesters, delay of radiation therapy until after delivery is possible, with the administration of chemotherapy in the meantime [25]. This approach is also used in the majority of non-pregnant patients receiving adjuvant chemotherapy, where radiotherapy is given approximately six months post-lumpectomy. In the first trimester, a breast conserving approach is more complicated due to the increased time of delay in radiotherapy, and the patient should be notified of the possible increased risk of local recurrence. Thus, mastectomy might be preferred. In addition, due to typical delay in the diagnosis of PABC, as discussed above, patients are frequently diagnosed with relatively large tumors requiring a radical approach. When mastectomy is the treatment of choice, breast reconstruction is possible [29]; however, due to the physiological changes during pregnancy, patients should consider postponing the procedure until after delivery.

While the American Society of Clinical Oncology (ASCO) does not recommend sentinel lymph-node biopsy (SLNB) in PABC patients [30], National Comprehensive Cancer Network (NCCN) guidelines approve this procedure during pregnancy as several studies showed it may be safely performed. Therefore, this procedure should be offered to pregnant patients over axillary dissection when indicated [31,32,33]. An estimated absorbed dose of between 0.1 mGy and 4.3 mGy [31,34] has been measured after an injection of 92.5 MBq of technetium 99m-labelled sulphur colloid into the mother breast, much less than the 100 mGy threshold. In addition, a 1-day protocol is advised for pregnant patients to minimize radiation exposure [4,25]. Although blue dye is not recommended for pregnant breast cancer patients due to possible anaphylactic maternal reaction, which in turn may cause fetal distress, recent study did not find any complications for seven pregnant women who received blue dye for mapping [35].

### 3.2. Radiation Therapy

Radiation therapy (RT) is generally not recommended during pregnancy and should be delayed until after delivery when possible (Figure 2). However, for non-pregnant breast cancer patients, it has been shown that adjuvant radiation initiated within 8–12 weeks after lumpectomy results in better DFS and smaller risk of local recurrence [36]. Therefore, when RT is absolutely indicated, it might be considered in highly selected patients, while reducing the fetal exposure to minimum [25,37]. The risks of fetal exposure to radiation vary according to gestational age and radiation dose. Generally, a radiation dose below 0.1–0.2 Gy is considered safe for the fetus [38]. Upon exposure to higher radiation doses, during the first two weeks after conception, failure to implant or undetectable death is expected [39]. In weeks 2–8 after conception, malformations may occur, especially in developing organs at the time of exposure. During weeks 8–25, the central nervous system (CNS) is extremely sensitive to radiation. Reduction in intelligence quotient (IQ) [40,41] and mental retardation are possible, where the risk increases with dose scatter to the fetus [42,43]. The risk for adverse events is decreased significantly after week 25 of gestation [38,39]. In addition, a general carcinogenic effect had been described for any in-utero radiation exposure at any gestational age, leading to higher risk of future malignancies in the offspring [37,44].

The radiation dose absorbed by the fetus depends greatly on the distance from radiation field, which in turn depends on fetal size and location, and hence on gestational age. The fetal radiation doses have been measured and calculated by several methods [45,46,47], where the calculated absorbed dose by an unshielded fetus did not exceed the threshold value of 0.1–0.2 Gy for therapeutic dose of 50 Gy, when given in the first trimester. Furthermore, appropriate shielding of the fetus may reduce by approximately 50–75% the doses from scattered radiation [37,48].

While several studies have reported the birth of a healthy child after RT for PABC [45,46,49], the long-term effect remains to be elucidated, thus it is generally considered as not recommended during pregnancy. However, RT is not an absolute contraindication for PABC and may be considered in highly selected cases following risk–benefit assessment with the mother, while considering gestational age, RT necessity, and treatment variables such as energy used, field size, shielding, etc.

### 3.3. Chemotherapy

Chemotherapeutic treatment is indicated in the majority of PABC cases, either in the adjuvant or neoadjuvant settings. During pregnancy, the same clinical guidelines should be implemented as in non-pregnant patients, while considering gestational age. In this context, the pregnancy timeline may be divided into four sections.

In the first 10 days after conception, an “all-or-none” event is expected—either a future development of the embryo or a miscarriage will occur.

The second period is from 10 days of conception until the end of the first trimester (14 weeks of gestational age). This period includes the organogenesis period, thus congenital malformations may occur after exposure to cytotoxic agents [50,51].

In the second and third trimesters, chemotherapy administration may result in IUGR, prematurity, low birth weight and myelosuppression [52,53]. However, recent studies suggest that the prevalence of such adverse events (AE) does not differ significantly from that of the common population [54,55,56]; thus, if indicated, chemotherapy should not be postponed throughout this period (Figure 2).

The last period is beyond 35 weeks of gestation, where administration of chemotherapy should be avoided as it may induce myelosuppression followed by AE at the time of delivery, such as bleeding, sepsis, or death (Figure 2). Further support to this recommendation was demonstrated when detectable levels of paclitaxel were found in the blood of a fetus 7 days, but not 21 days, after maternal administration [57].

Several chemotherapeutic regimens have been investigated for the treatment of pregnant breast cancer patients. No serious adverse consequences for the mothers or neonates were found when chemotherapy was administered in the second or third trimester [54,58]. Loibl et al. [13] found lower birthweight and more complications in infants exposed to chemotherapy in-utero compared to infants born to PABC patients that were treated with chemotherapy after delivery. However, these differences were not clinically significant and were most likely prematurity-related, thus avoiding premature delivery is recommended. A recent large international cohort study [14] found an interesting correlation between platinum-based chemotherapy and smaller size for gestational age and between taxane chemotherapy and neonatal intensive care unit (NICU) admission. The authors therefore recommend involving hospitals with obstetric high-care units in the management of these patients.

The relatively good tolerance of the fetus to maternal chemotherapy in the second and third trimesters may be attributed to limited exposure to the cytotoxic drugs compared to the pregnant mother. Observations in primates show that effectively all the drugs reach the fetus in a relatively low concentration, with variation between different agents [59,60].

It is well known that physiological changes during pregnancy (e.g., increase in plasma volume and increase in glomerular filtration rate) have an impact on the pharmacokinetics of therapeutic agents, including chemotherapy [61]. This fact raises doubt about the effectiveness of chemotherapy during pregnancy. Van Hasselt et al. [62] found a consistent decrease in exposure for doxorubicin, epirubicin, docetaxel, and paclitaxel. Thus, they do not recommend a priori dose reductions during pregnancy. Moreover, dose regimen adjustments might be considered for taxanes that showed larger decreases. However, to date, no evidence suggests that standard treatment in pregnant women is less efficient than in non-pregnant patients, and the same treatment regimen should be used for pregnant women [63], while adjusting the dose to the changes in body weight [23].

Dose-dense (DD) chemotherapeutic regimens (the same dose administered over a shorter interval) have been shown to improve DFS and OS in high-risk non-pregnant patients, particularly for women with HR-negative breast cancer [8]. However, data on the benefits and toxicities of these regimens during pregnancy are still lacking. No difference was found between PABC patients receiving DD chemotherapy and those receiving conventional chemotherapy in terms of pregnancy complications and congenital anomalies [64]. While this study included only a small cohort, it seems like DD chemotherapy is an acceptable option for PABC patients.

To conclude, chemotherapy for PABC is contraindicated in the first trimester and should be avoided also after 35 weeks of gestation. However, chemotherapy should be administered during the second and third trimesters, although close monitoring by a multidisciplinary expert team is advised.

### 3.4. Hormonal Therapy

**Figure 3:**
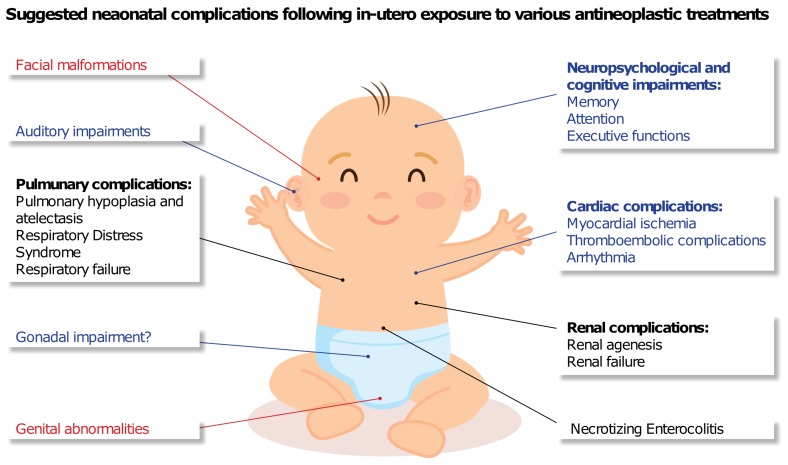
Possible documented neonatal outcomes following in-utero exposure to hormonal therapy (red) [65], targeted therapy (black) [72], and chemotherapy-related long-term outcomes (blue) [73,74].

Adjuvant endocrine therapy is administered in patients with HR-positive breast cancer. Tamoxifen, a non-steroidal selective estrogen receptor modulator, is the standard adjuvant endocrine treatment, given for 5–10 years depending on the disease features [65]. This endocrine regimen has been shown to reduce the risk of recurrence and mortality [66]. Currently, the use of tamoxifen is considered contraindicated during pregnancy (Figure 2), and it is recommended to stop tamoxifen at least two months before conception [67,68]. This recommendation is based on animal studies [65] and several case reports of birth defects including craniofacial malformations, ambiguous genitalia, and fetal death (Figure 3) [69,70,71]. In addition, to date, there is a paucity of information on long-term outcomes of the exposed infants. Such information could be of particular importance due to the biological similarity of tamoxifen to diethylstilbestrol, which is known to cause long-term adverse events.

Several reports have studied the effects of tamoxifen use during pregnancy. A recent study by Schuurman et al. [65] reviewed the literature and collected data of 167 cases of tamoxifen use during pregnancy with known pregnancy outcomes. They report a relatively high incidence of congenital abnormalities of 12.6% after tamoxifen exposure during pregnancy compared to 3.9% in the general population. However, the malformations described are non-specific, and most of the infants exposed to tamoxifen were born healthy. Moreover, confounders like concomitant medication or lifestyle reduce the causal relationship between tamoxifen and pregnancy outcome. The above, together with the possible disadvantages of postponing or discontinuing tamoxifen for the maternal prognosis, have led the investigators to conclude that patients should be counseled about the use of tamoxifen during pregnancy instead of blindly avoiding it; however, until additional supporting data are available, tamoxifen should be considered contraindicated during pregnancy, as stated above. An ongoing international multicenter trial (POSITIVE) may shed light on the consequences of postponing hormonal therapy. In this trial, the investigators aim to evaluate pregnancy, disease outcome, and safety of interrupting endocrine therapy to allow pregnancy for women with ER-positive breast cancer [75]. Results are expected by 2028.

### 3.5. Targeted Therapy

The incidence of HER2-positive tumors exceeds 20% of PABC cases [76]. Trastuzumab is the mainstay treatment of HER2-positive breast cancer [77,78]. However, currently, the administration of trastuzumab is not recommended for PABC patients (Figure 2).

A meta analysis study [72] included 18 patients with PABC treated with trastuzumab in the metastatic and adjuvant settings. The most common AE was oligohydramnios/anhydramnios, which occurred in 73.3% of the patients exposed to trastuzumab in the second/third trimester compared to none of those exposed in the first trimester. Moreover, all neonates exposed to trastuzumab in-utero exclusively in the first trimester were healthy at birth and at the long-term evaluation (median follow-up of nine months), compared to 57% of neonates with AE at birth (see Figure 3) and 25% in-utero deaths when trastuzumab was given during the second/third trimester. To note, there were no reports of fetal cardiotoxicity following in-utero exposure to trastuzumab during pregnancy.

The above observations may be attributed to the large molecular size of trastuzumab, which requires active transport across the placental barrier, a mechanism that does not exist early during pregnancy, leading to poor trans-placental transfer at this period [79]. Another hypothesis relates to the role of the fetal kidneys in producing amniotic fluid since the second trimester, where blocking epidermal growth factor receptor (EGFR) with trastuzumab may impair kidney function, decreasing amniotic fluid production.

In the adjuvant setting, there is no evidence supporting the administration of trastuzumab during pregnancy, since trastuzumab may be also effective when given after six months of adjuvant chemotherapy [77]. Thus, it may be delayed until after delivery. However, in the metastatic setting, when urgently needed, it can be given for a short period of time, while monitoring amniotic fluid level and fetal growth. The treatment should be discontinued immediately in any signs of oligohydramnios, as this process has been proven to be reversible.

Lapatinib is an oral anti-HER2 and anti-HER1 tyrosine kinase inhibitor, which was found not cross-resistant with trastuzumab in preclinical trials [80]. Lapatinib as a single agent or in combination with trastuzumab is approved for the treatment of patients with HER2-positive metastatic breast cancer [81,82] and has recently been shown to increase the pathologic complete response in the neoadjuvant setting (NeoALLTO trial) when combined with trastuzumab [83]. However, in the adjuvant setting (ALLTO trial), no improvement in DFS was found and toxicity rates were higher [84]. In both the NeoALLTO and ALLTO trials, pregnancy data and outcomes were prospectively collected [85]. A total of 12 patients were exposed to trastuzumab and/or lapatinib during gestation or up to seven months (trastuzumab) or seven days (lapatinib) prior to pregnancy. Only five women completed their pregnancy, where the rest had induced abortion, and all these women were exposed before or during first trimester only. No complications were noted during pregnancies or deliveries in these patients, and all pregnancies resulted in live births without congenital anomalies. Despite the small cohort, these results further support the recommendations above that pregnancy could be continued when treated with anti-HER2 agents in the first trimester.

Recent data suggest the benefit of treatment with pertuzumab in addition to trastuzumab and chemotherapy in the neoadjuvant, adjuvant, and metastatic settings in HER2-positive breast cancer [86,87,88,89]. However, there is a paucity of data on pertuzumab in pregnant patients. A recently published case report of a pregnant woman treated with trastuzumab and pertuzumab for metastatic breast cancer until 20 weeks of gestation showed oligohydramnios which evolved into anhydramnios, right renal agenesis and fetal growth retardation, resulting in pregnancy termination [90]. An ongoing observational prospective trial (MotHER) studies the pregnancy and pregnancy outcomes in women with breast cancer treated with trastuzumab, pertuzumab in combination with trastuzumab or ado-trastuzumab emtansine, and results are pending [91]. Until additional data are available, pertuzumab is not a recommended treatment during pregnancy as well.

To conclude, targeted therapy should be postponed until after delivery. If strongly indicated, trastuzumab may be given in the metastatic setting for a short period of time with close monitoring, where lapatinib and pertuzumab should be avoided completely until further data are available.

### 3.6. Immunotherapy

Immunotherapy in the treatment of breast cancer is promising, although not yet mature. Clinical trials imply that triple-negative and HER2-positive breast cancer, which are more common in PABC patients as discussed above, express high immunogenicity, and response to checkpoint inhibitors has been shown, especially when combined with standard therapies [92,93]. While initial results are promising, only about 20% of treated breast cancer patients showed response, hence predictive markers are still needed to identify the subset of patients for which the treatment will benefit the most.

During pregnancy, the mother develops immune tolerance to the fetus. Immune checkpoints have an important role in this process and hypothetically inhibition of these checkpoints may result in an immune response against the fetus. Studies in animal models showed association between immune checkpoint inhibitors and increased rates of abortions, stillbirths, premature delivery, and infant mortality; however, no human trials have been done thus far [94]. Until sufficient data on the safety of immunotherapy during pregnancy is collected, this treatment is contraindicated during pregnancy.

## 4. Obstetrical Care

In general, PABC patients should be considered as high-risk obstetric patients, with routine fetal and maternal health checkup at least once every three weeks [95]. Fetal development should be assessed prior to the initiation of treatment. As discussed above, a time-interval of three weeks is recommended between last chemotherapy administration and delivery and preterm delivery should be avoided. Vaginal delivery is preferred since post-partum anti-neoplastic treatment can be resumed immediately after delivery, while at least a one-week interval is recommended after caesarean section. After delivery, the placenta should be pathologically inspected as previous reports found placental metastases [96,97]. Breastfeeding during and a few weeks following chemotherapy administration is not recommended, and milk production should be inhibited to avoid accumulation of lipophilic anti-neoplastic agents in the milk [4].

## 5. Long-Term Neonatal Outcomes

Long-term effects following in-utero chemotherapy exposure is a major concern and should be taken into consideration in risk–benefit analysis of any PABC patient [2,98]. Several studies have been conducted to evaluate the long-term effects, focusing mainly on neurological, psychological, cognitive and general developmental, as well as cardiac side effects (Figure 3). The main results from these studies are listed in Table 1.

All studies on long-term neonatal outcomes following in-utero exposure to chemotherapy report normal development, educational performance and neurocognitive behavior with no congenital, neurological or psychologic abnormalities in exposed children compared to non-exposed population [55,73,99,100,103,104,105]. As cardiac function is also a major concern in exposed infants, several studies have examined the effect of in-utero exposure to chemotherapy on cardiac function and measures; however, no cardiac abnormalities or dysfunction was found [73,101,102,105]. Murthy et al. [103] report no significant delays in puberty in 50 children with a median age of seven years who were exposed to FAC in-utero. The authors also note that allergies and/or eczema occurred more commonly in exposed children. Amant et al. [73] report normal CNS and auditory function in 70 children who were exposed in-utero to chemotherapy. In addition, they observe prematurity-associated lower cognitive development scores when compared to the general population. Another study by Amant et al. [105] compared 129 children to mothers who were diagnosed with cancer during pregnancy to children born to mothers without cancer. They report correlation between prematurity and worse cognitive outcomes; however, this result was independent of cancer treatment. The results from these two studies imply that prematurity, rather than in-utero exposure to cytotoxic agents, is responsible for the cognitive impairment, in line with the normal neurocognitive behavior observed in other studies, as discussed above. Therefore, avoiding iatrogenic preterm delivery is recommended when possible.

Figure 3 illustrates potential neonatal outcomes that had been proposed in the clinical and preclinical setting. As described above, most of the studies found no significant clinical neonatal adverse outcomes in children exposed in-utero to chemotherapy. However, long-term outcomes are still a major concern after in-utero exposure to anti-neoplastic treatment. Additional long-term case-control and prospective studies are still needed to fully determine the implications of in-utero exposure and to better evaluate the risk–benefit of each treatment.

## 6. Conclusions

Cancer associated with pregnancy represents a unique clinical scenario that requires a delicate balance of risks and benefits for both maternal and fetal well-being, as well as a multidisciplinary discussion and close monitoring by an expert team. To date, there is a paucity of data regarding the short- and long-term outcomes of in-utero exposure to radiation and anti-neoplastic agents. In the diagnosis procedure, special care should be given to minimizing fetal radiation exposure. Thus, staging examinations should be performed only if results may alter clinical management. In general, it is not advised to postpone surgical and chemotherapeutic treatment, as delaying treatment might worsen maternal prognosis. However, fetal risks must be considered and chemotherapy should not be given before the second trimester and in the vicinity of labor. Individual risk–benefit assessment should be undertaken, while considering various factors such as disease stage, gestational age, maternal and fetal risks and alternative treatment options, in order to determine the necessity of other therapeutic modalities such as radiotherapy and targeted therapy, where postponing these until after delivery is advisable. Aside from an emergency situation, iatrogenic preterm delivery is not recommended as prematurity increases the rate of long-term abnormalities and should be avoided.

Current evidence, as comprehensively reviewed herein, still represent retrospective data with a substantial body of evidence that is based upon case reports. Moreover, the clinical outcomes in several of the studies are based on crude assessments that may not reflect subtle changes in neurocognition function, cardiac function, and future impact on fertility due to the potential gonadotoxic effect to the evolving gonads during in-utero exposure. Revealing the mechanism of chemotherapy-induced placental toxicity may shed light on the potential pathway that may mediate the subsequent sequel to the embryo. Further studies should also emphasize the long-term outcomes of in-utero exposure to anti-neoplastic treatments that are crucial for better risk–benefit analysis. In view of the rarity of PABC, prospective and randomized control trials are difficult to implement, hence international multi-centric registries for both maternal outcomes and offsprings long-term follow up are crucial and should provide sufficient data for better management of PABC.

## Figures and Tables

**Table 1:** Long-term neonatal outcomes following in-utero exposure to chemotherapy.

Study	Malignancy	No. of Patients	Exposure Trimesters	Follow-up Duration	Outcomes
Nulman et al. (2001) [99]	Various	106	1T/2T/3T	1 month– 22 years	Normal Development and school performance
Aviles et al. (2001) [100]	Hematologic	84	1T/2T/3T	18.7 (6–29) years	Normal learning and educational performance. No congenital, neurological or psychologic abnormalities. No malignancies reported.
Aviles et al. (2005) [101]	Hematologic	81	1T/2T/3T	17.1 (9.3–29.5) years	Normal echocardiogram. No cardiac toxicity or dysfunction.
Van Calsteren et al. (2006) [102]	Various	10	2T/3T	2–66 months	Full neurological and cardiological examination found no abnormalities. Cortical malformation in a twin.
Hahn et al. (2006) [55]	Breast	40	2T/3T	2–157 months	Normal development. Normal health. Normal school performance. 1 ADD. 1 Down syndrome.
Amant et al. (2012) [73]	Various	70	2T/3T	22.3 (16.8–211.6) months	Normal growth and general health. Normal cardiac, CNS and auditory function. Normal neurocognitive development. Subtle changes in cardiac measurements were noted. Prematurity-associated lower cognitive development scores.
Murthy et al. (2014) [103]	Breast	50	2T/3T	7 (<1–21) years	No significant toxic effects. Normal development. No significant delays in puberty. Allergies and/or eczema with higher prevalence.
Cardonick et al. (2015) [104]	Various	57	2T/3T	18–124 months	Maternal cancer, exposed vs. unexposed to chemotherapy. No differences in: Cognitive skills. Academic performance. Behavioral competence.
Amant et al. (2015) [105]	Various	129	2T/3T	Follow-up at 18 and 36 months	In-utero exposure to various treatment types vs. matched control group (no treatment). No difference in cognitive and general development. Cardiologic evaluation at 36 months showed normal findings. Prematurity was correlated with a worse cognitive outcome independent of cancer treatment.
O’Laughlin et al. (2019) [58]	Breast	26	2T/3T	Mean 44 months	Comparison between in-utero exposure to chemotherapy + taxanes and chemotherapy alone. No difference in developmental or behavioral assessments. No medical disorders diagnosed.
